# Estimating the incidence of colorectal cancer in South East Asia

**DOI:** 10.3325/cmj.2013.54.532

**Published:** 2013-12

**Authors:** Inka Kokki, Angeliki Papana, Harry Campbell, Evropi Theodoratou

**Affiliations:** 1 Centre for Population Health Sciences, University of Edinburgh, Edinburgh, UK; 2 Department of Economics, University of Macedonia, Thessaloniki, Greece; * The first two authors contributed equally.

## Abstract

**Aim:**

To estimate the burden of colorectal cancer (CRC) in South East Asia.

**Methods:**

We reviewed the evidence from the published literature found through a systematic review in Medline, Embase, and Global Health and from unpublished data on cancer registries, which were sourced from the International Agency for Research on Cancer. Incidence rates were summarized by calculating descriptive statistics and meta-analysis estimates.

**Results:**

The crude mean incidence of CRC in South East Asia for both sexes was 6.95/100 000 population and the incidence increased with age. The crude meta-analysis estimate was 6.12/100 000 population (95% confidence interval 5.64-6.60/100 000) and the number of new CRC cases for 2000 was 32 058 (29 544-34 573).

**Conclusion:**

The rates of CRC in South East Asia were much lower than those reported for high-income countries, but higher than those reported for Sub Saharan Africa.

The International Agency for Research on Cancer (IARC) estimated that approximately 1.2 million new cases of colorectal cancer (CRC) were diagnosed in 2008 (9.8% of all new cancer cases) making CRC the fourth most common cancer worldwide (1). In addition, CRC accounted for over 600 000 of the 7.6 million cancer deaths. Large variations in incidence rates were observed, with the lowest incidence rates reported in regions of central Africa and south-central Asia and the highest in western regions of Europe, North America and Australia/New Zealand (1).

Although CRC is mainly a disease of the high-income countries, there has been a rapid increase in rates of low and middle income countries that have recently made the transition from a relatively low- or middle-income economy, such as Japan, Singapore, and eastern European countries (2). This is also reflected by the fact that the percentage of the new CRC cases recorded in the more developed regions dropped from 65% in 2002 to 59% in 2008 (1). These changes are partly due to the aging population of low and middle income countries but also due to the link of CRC with several dietary factors and lifestyle habits. In particular, several components of the diet have been linked with increased (red and processed meat intake) or decreased (dietary fiber, fruit and vegetables, vitamin D) CRC risk (3-7). In addition, lifestyle habits like high energy intake, increased body weight, low physical activity, smoking and high alcohol intake have been found to be associated with increased CRC risk (7).

In high income countries, the main source of cancer data are cancer registries. Generally, cancer registries collect, maintain, and manage cancer data and they are unique in being able to monitor changes in cancer incidence and survival over long periods of time (8). However, they cover less than 25% of the world’s population and it is estimated that this proportion would reduce to 11% if only data of good quality were included (9). Currently, both the World Health Organization (WHO) and the IARC collect data on cancer deaths from cancer registries and produce estimates of the global and regional burden of cancer. In particular the IARC publishes its sets of estimates of global cancer incidence and mortality through the GLOBOCAN project, with the most recent one being from 2008 (1).

The South East (SE) Asia region includes the following 11 countries based on the WHO classification: Bangladesh, Bhutan, Democratic People’s Republic of Korea, India, Indonesia, Maldives, Myanmar, Nepal, Sri Lanka, Thailand, and Timor-Leste. The 2008 GLOBOCAN data based on cancer registries estimated that CRC was the 4th most common cancer in SE. Asia, with an age-standardized incidence rate of 6.9/100 000 and accounting for almost 100 000 CRC cases (6% of all cancers in SE. Asia) (1). However, the reliability of the information provided in SE. Asia cancer registries is open to question with only 5 of the 11 SE. Asian countries having a formal registration system for cancer (Bangladesh, India, Indonesia, Myanmar, and Thailand) (10).

In this review, we estimated the burden of CRC in the SE. Asia region. We reviewed the evidence from the published literature found through a systematic review and unpublished data on cancer registries and we also compared the estimated CRC burden in SE. Asia with that estimated from a similar exercise in Sub-Saharan Africa (11). Finally, we aimed to explore the quality and availability of data of cancer registries and to make suggestions for research and public health policy priorities to improve control of CRC.

## Methods

The data concerning colorectal cancer in SE. Asia were collected from published papers through a systematic review of the literature, and from unpublished cancer registry data. Data from the literature were further divided as registry based and hospital based in order to compare the results of the analysis based on the data source.

### Search strategy for systematic analysis and data extraction

The search was conducted through OvidSP, using Embase, Global Health, and Medline as the resources. The search terms for the systematic data extraction from the published papers are presented in Supplementary Table 1(supplementary table 1). We included primary research articles, limited to post-1980 that were conducted in SE. Asia region, as defined by WHO. The studies involved any age-group or sex and there was no restriction on the language of publication, as long as a translation into English was available for the full article. Studies were excluded if they did not present clear, numerical information on the colorectal cancer incidence, prevalence, and/or mortality or did not present a clear case definition of colorectal cancer.

The initial search of the published articles produced 1138 results from the three databases (Embase, Global Health, and Medline), while this number was reduced to 734 results after cross-checking the results, removing duplicates, and limiting the publishing year to post-1980. Consequently, the article titles were read to do the first exclusion from the results, leaving 59 abstracts and full articles to read. Abstracts not fitting the inclusion criteria, mostly due to wrong location of study or population, were removed. After reading the full texts, there were 31 studies left to be included. Two of these were duplicates of two articles, published in separate journals, so they were also excluded, as well as a review article, leaving 28 articles to be included. Six more articles were recovered based on the references of the 28 articles. Therefore, from the systematic search, 34 papers related to colorectal cancer in SE. Asia were found, with publication dates ranging from 1984 to 2012. In this review, we only examined the incidence of CRC and therefore the final number of the included published papers was further reduced to 13 (12-24) (Supplementary Figure 1)(supplementary figure 1).

Along with the systematic review, registries in countries belonging to the IACR Asian region and in countries of the SE. Asia region were contacted in an attempt to get additional data. In total 26 registries from 5 countries were contacted, but this has proved mostly unsuccessful and we only got one reply. However, registries from India, Thailand, and Sri Lanka had accessible publications online, and therefore we were able to extract the required information concerning the incident rate of CRC, avoiding duplicate results.

### Data analysis

This review included 43 data points available for CRC from the published articles and 103 data points from the registries. This provided a total of 146 data points, 90 for the calculation of incidence rate of CRC, 30 for colon cancer, and 29 for rectal cancer. Incidence estimates were either extracted from the included data sets or calculated using the data reported. All estimates were converted to incidence per 100 000 of population per year to allow for direct comparison between results. To estimate the mean (standard deviation) and median (1rst quartile Q_1_-3rd quartile Q_3_) of the CRC incidence rate in SE. Asia we considered all the data points together and by publication type (unpublished data that have been found from registries or from published papers) and by data source (data from cancer registries or hospital based data). In addition, a meta-analysis was conducted to estimate the pooled incident rate and corresponding 95% confidence interval (CI) using both fixed and random effect models (Mantel-Haenszel and DerSimonian-Laird methods, respectively).

Data sets separated in age groups were also included in our study. A total of 640 data points were available for the age-group analysis, the majority of which were extracted from cancer registries. The age ranges were based on the groups used by the data sets from the IACR. From this, the minimum, maximum, lower quartile, upper quartile, mean, median, and variance were calculated for each age group and box-plots are presented to summarize the information from these data. Analyses were conducted using STATA 12.0 (Statacorp, College Station, TX, USA) and SPSS 21.0 (SPSS Inc., Chicago, IL; USA) statistical software.

## RESULTS

**Figure 1:**
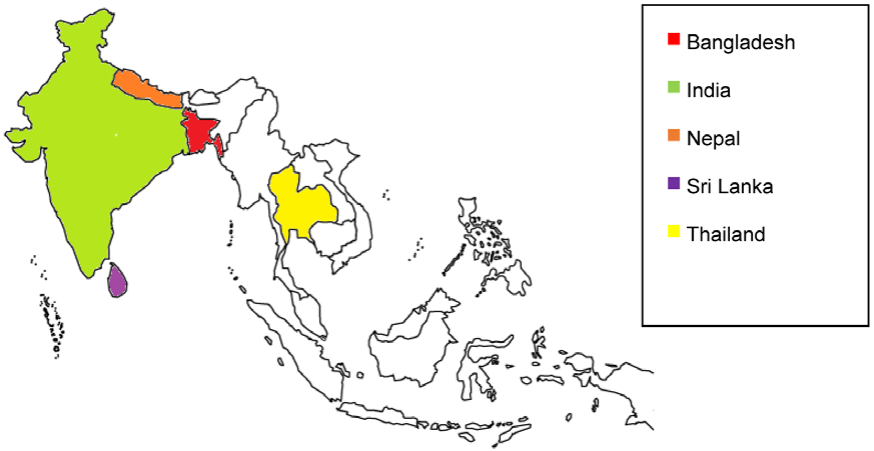
Geographical distribution of the included data sets.

The data sets both from the published literature and from the cancer registries came from five countries of the SE. Asia – Bangladesh, India, Nepal, Sri Lanka, and Thailand (Figure 1). We report the results for colorectal cancer and in the supplementary document we present the results separately for colon and rectal cancer. The majority of the data were from India and Thailand, ie, 35.6% of the data came from India, 55.6% from Thailand, 4.4% from Sri Lanka, 3.3% from Nepal, and 1.1% from Bangladesh. Furthermore, 74.4% of the data was extracted from cancer registries (unpublished), while the remaining was from published papers. Furthermore data from published papers were either from registries (19/43 = 44.2%) or hospitals (24/43 = 55.8%). Data sets from Nepal and Bangladesh came solely from the published papers.

Clusters of data sets came from larger cities (eg, New Delhi, Mumbai, and Kolkata) and all data sets reported CRC from all age groups. The data sets covered the period 1980-2011, with the majority of studies referring to the period 2001-2003 (21.1%), 2004-2006 (20%), 1998-2000 (11.1%), 1997-1998 (6.7%), 1995-1997 (6.7%), and 1990-1996 (6.7%). (Supplementary Figure 2**)**(supplementary figure 2).

### Incidence of CRC in SE. Asia

All data sets included information about the years of the study, the number of cases considered, and the population size. The years of study varied from 1 to 10, with the majority of studies considering 3 years (60%), 1 year (16.7%), and 2 years of study (7.8%). The number of cases considered varied from 3 to 12 486 cases (the mean number of cases was 715), and the population varied from 14 550 to 59 594 155 (the mean population was 6 350 312)(Supplementary Figure 3 (supplementary figure 3) and Supplementary Figure 4 (supplementary figure 4)**).**

**Figure 2:**
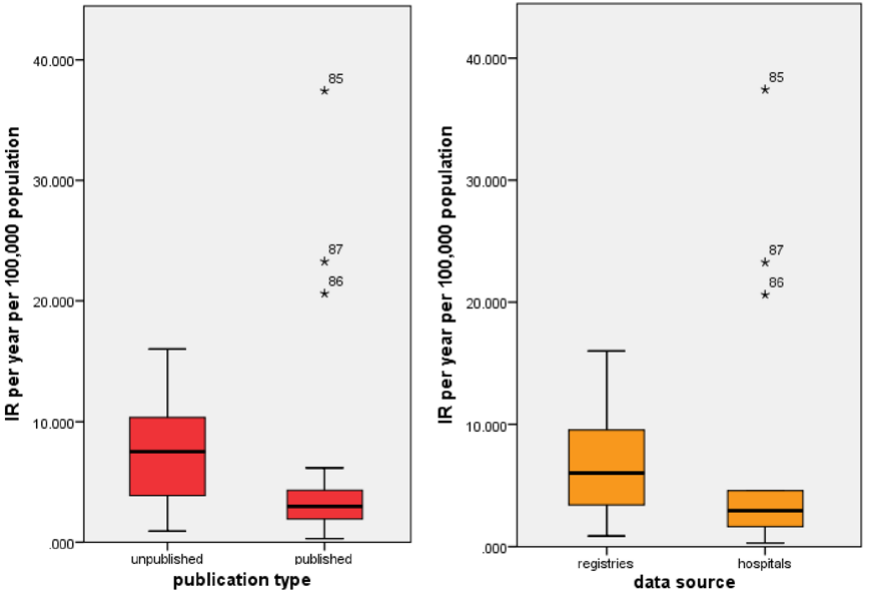
Boxplot of incidence rate of colorectal cancer per year per 100 000 population regarding the publication type (published or unpublished) and the data source (cancer registries or hospitals).

**Figure 3:**
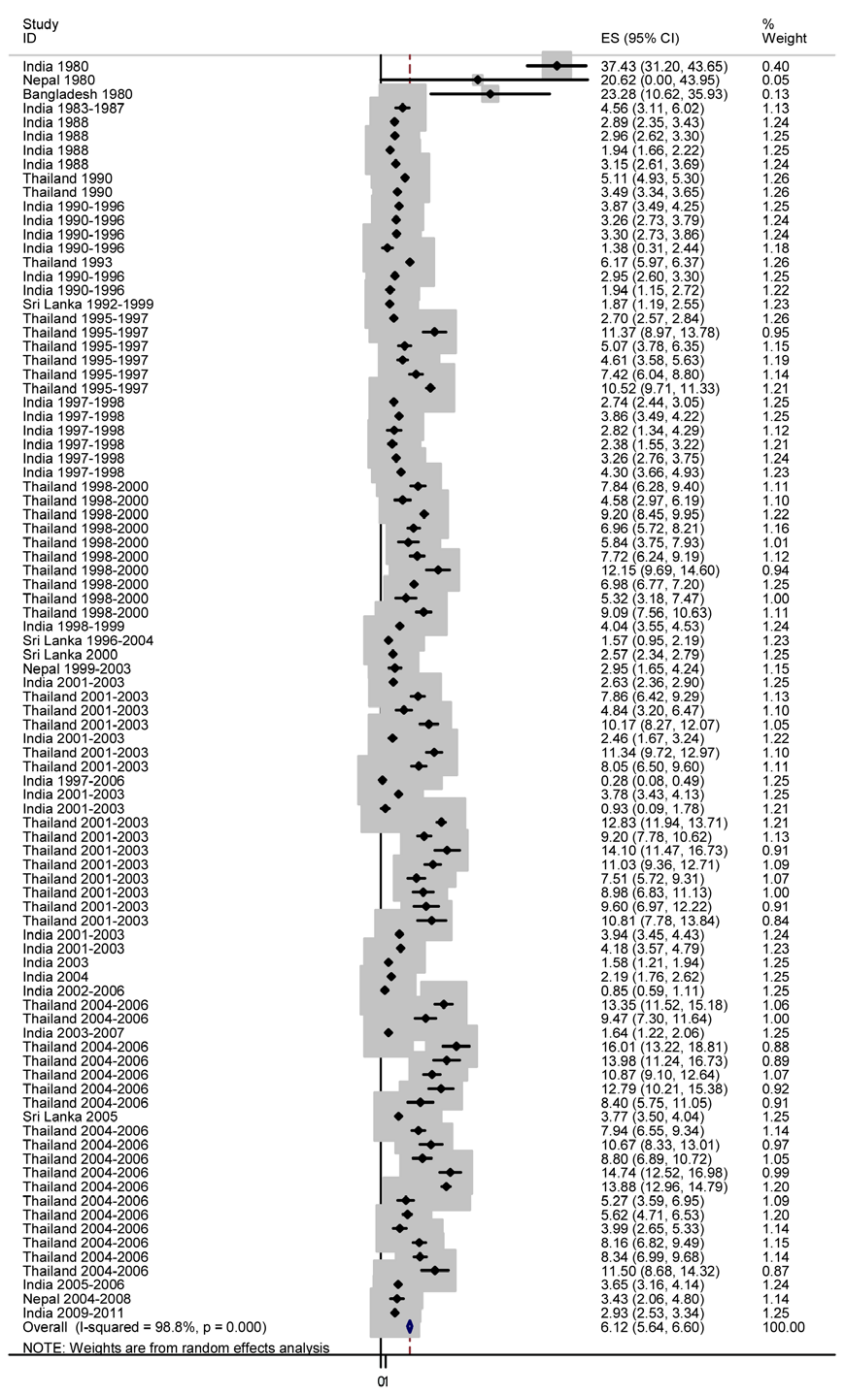
Meta-analysis of the incidence rate per year per 100 000 population of colorectal cancer

**Figure 4:**
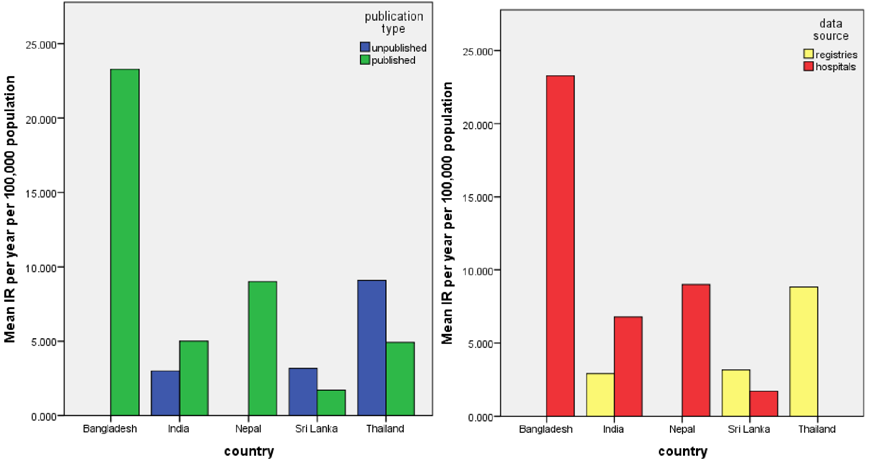
Mean incidence rate of colorectal cancer per year per 100 000 population for each country (Bangladesh, India, Nepal, Sri Lanka, and Thailand), regarding the publication type or the data source.

The incidence rate per year per 100 000 population ranged from 0.285 to 37.43, while the majority of the incidence rates were below 10 (Supplementary Figure 5)(supplementary figure 5). The mean (SD), median (Q_1_-Q_3_) and meta-analysis estimate (95% CI) for the crude annual incidence rate are presented for all data and separately by publication status (published data and data from cancer registries) and by data source (cancer registries and hospital derived data) (Table 1). The mean annual crude incidence rate for all data combined was 6.96/100 000 population and the meta-analysis estimate based on random effects model was 6.12 (95% CI 5.64-6.60) (Table 1, Figure 2 and 3). When we compared the incidence rates by publication type, the mean incidence rates from the published papers (6.02/100 000 population) and the unpublished ones (7.28/100 000 population) did not differ significantly (*P* = 0.52, *t* test). Considering the data source, the mean incidence rate from the registries was 6.82/100 000 population and from the hospital based data was 7.71/100 000 population and they did not differ significantly (*P* = 0.77, *t* test). The highest incidents rates of CRC were observed in Bangladesh (hospital based data), while the lowest ones were observed for India and Sri Lanka (data were both from registries and hospital based) (Figure 4).

**Table 1:** Statistics for the crude annual incident rate of colorectal cancer (per year per 100 000 population), the number of cases and the population size. The number of data points is denoted by N (SD – standard deviation, IR – incidence rate, CI – confidence interval)

		Number of cases		Population		IR		IR	
Colorectal cancer	N	mean (SD)	Median (Q_1_-Q_3_)	mean (SD)	median (Q_1_-Q_3_)	Mean (SD)	Median (Q_1_-Q_3_)	Meta-analysis (95% CI)	I^2^ (95% CI)
**All data**	90	715.46 (1507.54)	297 (145-544.5)	6,350,312 (13,135,119)	1,553,538 (768,715-5,884,788)	6.95 (5.56)	5.19 (2.95-9.50)	6.12 (5.64-6.60)	99% (99-99)
**Publication status:**									
systematic review	23	530.35 (951.39)	183 (100-294)	1,054,915 (18,519,735)	4,130,000 (828,161-7,000,000)	6.02 (8.88)	2.96 (1.87-4.56)	3.46 (2.58-4.34)	99% (99-99)
cancer registries	67	779 (1657.38)	336 (192-614)	4,910,373 (10,492,508)	1,463,495 (753,241-4,452,434)	7.28 (3.87)	7.51 (3.86-10.52)	7.03 (6.43-7.63)	98% (98-99)
P*						0.517	0.001		
**Data source:**									
hospital based	14	165.50 (152.45)	129.50 (72-214)	2,410,214 (2,295,942)	1,560,000 (596,734-4,572,876)	7.71 (11.14)	2.94 (1.62-8.58)	3.14 (2.21-4.07)	97% (96-98)
cancer registries	76	816.76 (1620.50)	335 (185.25-616.25)	7,076,119 (14,155,981)	1,540,141 (776,199-6,245,066)	6.82 (3.88)	6.00 (3.35-9.57)	6.50 (6.00-7.00)	99% (99-99)
P*						0.772	0.032		

*P-values were calculated using *t* test for mean values and Mann-Whitney test for median values.

### Incidence of colon and rectal cancer in SE. Asia

The incidence rate per year per 100 000 population for colon cancer ranged from 0.123 to 3.494 and for rectal cancer from 0.162 to 2.87. The mean (SD), median (Q_1_-Q_3_), and meta-analysis estimate (95% CI) for the crude annual incidence rates of colon and rectal cancer were calculated. The mean annual crude incidence rate was 1.34/100 000 population for colon cancer and 1.62/100 000 population for rectal cancer (Supplementary Table 2 (supplementary table 2) and Supplementary Figure 5 (supplementary figure 5), Supplementary Figure 6 (supplementary figure 6), and Supplementary Figure 7 (supplementary figure 7) ).

### Incidence of CRC in AE Asia from different age groups

A total of 640 data points divided into 16 age groups were considered for the age-specific incidence of CRC. The majority of the data came from cancer registries (97.5%) and data were gathered from three countries: India (47.5%), Thailand (47.5%), and Sri Lanka (5%) (Supplementary Figure 8 (supplementary figure 8)**)**.

The data covered the period 1990-2006 and stemmed from 1 year (7.5%), 2 years (15%), 3 years (62.5%), and 7 years (15%) of study. The number of cases considered at the data sets varied from 0 to 780 cases. The age-related distribution of incidence showed a substantial increase with age. There were large variations reported mainly in the older age groups, whereas for younger age groups, small variations were observed.

**Figure 5:**
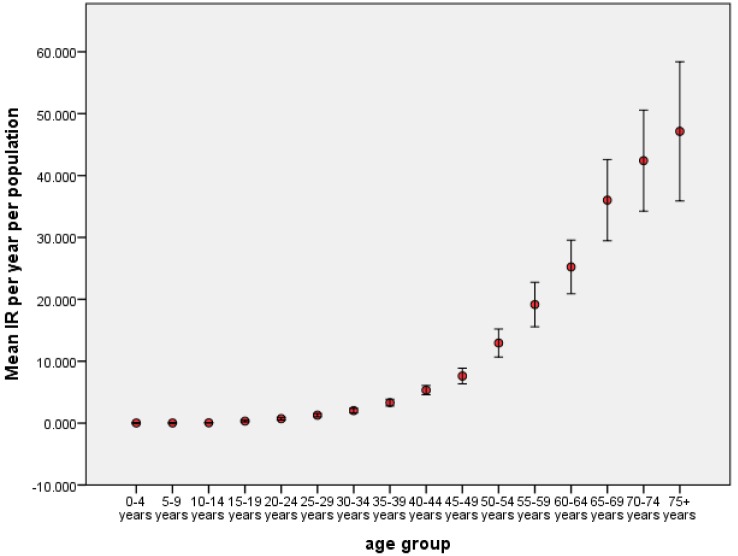
Mean incident rate (per year per 100 000 population) of colorectal cancer by age groups. Error bars indicate the 95% confidence interval.

The mean, median, and standard deviation of the incident rate of CRC increased with the age group, as expected. The mean incident rate was consistently slightly larger than the estimated median for all age groups, indicating that the distribution of the incident rate had a positive skew (skewed to the right) (Table 2, Figure 5 and Supplementary Figure 9 (supplementary figure 9) ).

**Table 2:** Statistics (mean, median, and standard deviation) for the incident rate per year per 100 000 population of colorectal cancer by age group

Age groups	0-4	5-9	10-14	15-19	20-24	25-29	30-34	35-39	40-44	45-49	50-54	55-59	60-64	65-69	70-74	75+
**Mean**	0.0236	0.0294	0.0552	0.3242	0.7177	1.2783	2.0229	3.3105	5.3249	7.6067	12.9466	19.1601	25.2275	36.0185	42.4071	47.1406
**Median**	0.0000	0.0000	0.0000	0.1870	0.5590	1.1620	1.6555	2.8180	5.1465	6.9400	11.1655	17.1610	20.5305	30.8330	38.1790	35.8390
**Standard deviation**	0.0912	0.1114	0.1122	0.4100	0.6496	0.8015	1.1525	1.7017	2.3133	3.9273	7.0879	11.2500	13.5276	20.5065	25.5481	35.1429

**Figure 6:**
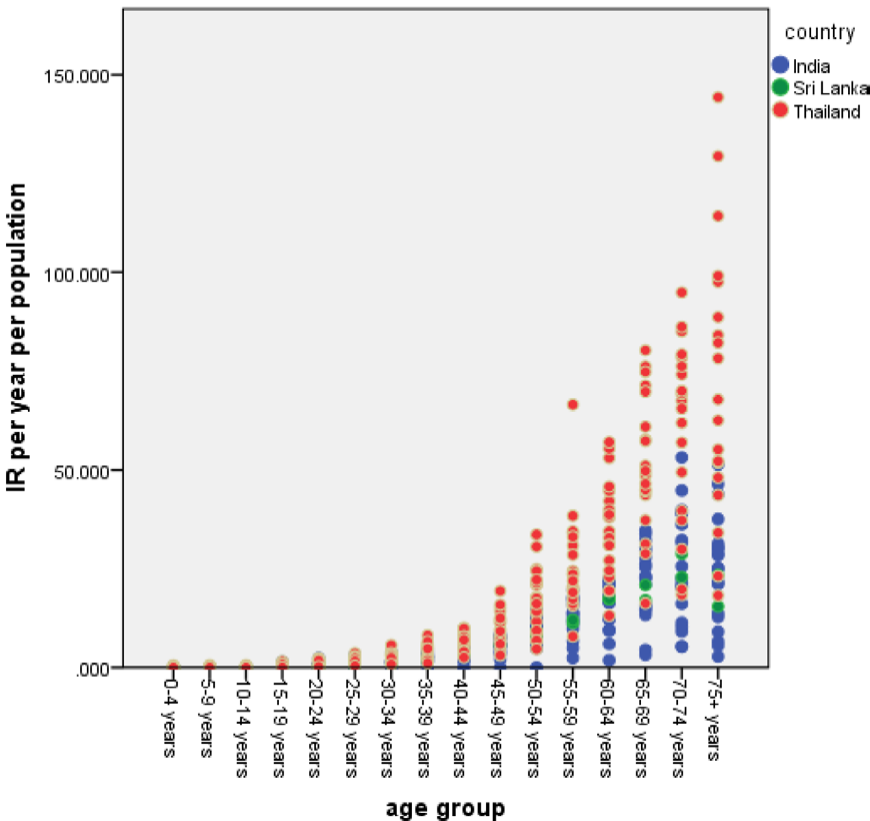
Incident rate per year per 100 000 population of colorectal cancer by age groups for each country.

When we examined the incident rate by the age groups for each country separately, it was again obvious that the incident rate increased with age, while data indicating the existence of CRC for ages below 40 came strictly from Thailand. The CRC patients for India come only from age-groups over 40, while for Sri Lanka from age-groups over 55 (Figure 6).

## DISCUSSION

In the SE. Asian region a mean incidence rate of CRC was 6.95 per year per 100 000 population, with age-specific rates ranging from a mean of 0.02 in the under 4-year-old group to 47.14 in the over-75-year-old group. When colon and rectal cancer were looked at separately, the mean incidence rates were 1.34 and 1.62 per year per 100 000 population, respectively. The rate derived from the meta-analysis for all CRC in the region was 6.12 per year per 100 000. The meta-analysis also showed that cancer registries had a tendency to produce higher rates than hospital based studies, although this difference did not always reach statistical significance. This was also observed in a review estimating the CRC incidence rate in Sub-Saharan Africa (11). Several reasons might explain this difference. Hospital based studies will usually cover people who have presented or referred to centers where there are cancer services as symptomatic, and who can access these health care facilities, both physically and financially.

The majority of the included data were generated in the late 1990s to early 2000s, with very little data available for the 1980s and a limited number from the last decade. As a result, we estimated the number of CRC cases for the year 2000. When we applied the incidence rate from the meta-analysis to the 2000 population of SE. Asia (25), this translated to 32 058 new colorectal cancer cases for the year 2000 (95% CI 29 544-34,573; based on the meta-analysis estimate).

The IACR publication GLOBOCAN from 2008 (1) reported an age-standardized incidence rate of 6.9/100 000, which supports our results. The incidence rate in SE. Asia was significantly higher when compared to the estimates for Sub-Saharan Africa (11), where the reported crude incidence rate was 4.04 cases per 100 000 population. When compared to a high income country such as the UK, the incidence rate in SE. Asia was many times lower, where the age standardized incidence rate for CRC was 45.3 per 100 000 population in 2000 (Cancer Research UK). This difference between CRC incidence in SE. Asia and high income countries might be due to underreporting and also due to poorer quality of cancer registration. However, since CRC is closely linked to poor diet, obesity, lack of exercise, and smoking these differences might also be true.

The availability of the data from cancer registries was beneficial to this review, but an aspect that could have further strengthened it would have been replies from other cancer registries with no freely available data. Twenty six registries were contacted via email, and only one replied, with many of the emails for registries on the IACR list for the region being outdated. In addition, the registry that replied was not updated with the latest figures so they were unable to provide any new information.

One of the caveats of this systematic review is the limited availability of published or cancer-registry data from certain countries in SE. Asia, such as the Democratic People’s Republic of Korea, and the high volume of data from few countries such as India and Thailand. This may reflect the fact that these countries have cancer registries and have a more organized data collection, whereas some countries in the same region are behind on these developments. On the other hand, many of the cancer registries only cover small regions within a country. In SE. Asia in particular, clusters of data sets came from larger cities like New Delhi, Mumbai, and Kolkata which are known to conduct high levels of research and where only 40% of the population lives. The prevalence of CRC risk factors in large cities might be higher and therefore the estimates may be exaggerated or under-representative of rural areas.

The quality of data in relation to publication bias could be improved in a couple of ways. First of all, more sources could be used to search for published literature, and the search could be widened to include more foreign-language articles. Journals from the countries that did not provide much data could be searched for information, but this might require different access and more translations for these journals. In addition, further attempts to contact registries through other means or updated emails could be attempted. One of the problems with the data from countries lacking cancer screening or registries is that they only pick up a portion of the population with CRC. This can be improved by developing such practices in these countries.

The incidence rate estimates presented in our review for 2000 and in GLOBOCAN for 2008 are very similar, despite the fact they had a different time frame. We believe that the lack of change in the CRC incidence rate between 2000 and 2008 is mainly due to the use of the same data (ie, lack of more recent data), rather than because the rates have stayed stable throughout the last two decades. This is supported by the fact that incidence of cancer, other non-communicable diseases, and risk factors in low and middle income countries is on the increase as these countries continue to develop and acquire new risk factors (26).

The increase in cancer incidence will put further pressure on the health systems of SE. Asia that are not yet adjusted to diagnosing and managing patients with this condition (27). Already the quality of cancer control in low and middle income countries is not adequate, where there is lack of human resources, physical resources, and equipment (28). For example in 12 countries in the Asia-Pacific region machine supply relevant to cancer treatment was sufficient to meet an estimated 23% of the current need (29) and only 43% of the SE. Asia countries had access to drugs for cancer therapy (28). In addition, the outcome of cancer services in low and middle income countries is poorer than in high income countries, with five-year survival rates being some times as half in developing countries (30). Therefore, it is clear that investments in health systems are required and this includes developing a supply of trained oncology professionals, treatment equipment, and cancer drugs for cancer prevention and control.

In summary, this systematic review has highlighted the lack of data on CRC, mirroring the lack of data for CRC in Sub-Saharan Africa (11). There is a notable lack of data for the last decade and also a complete lack of data for a number of SE. Asia countries, which limits the representativeness of the review. Furthermore, communication between cancer registries was not very successful and complete use of the existing data was not possible. Non-communicable diseases, including cancer, are increasing in low-income regions and therefore it is important that the burden of disease is accurately and regularly monitored. Therefore there is a great need to improve the volume and quality of information available on cancer in SE. Asia and generally in low and middle income countries and there needs to be stronger investment in the existing cancer registries as well as development of cancer registries in countries that are lacking one. Apart from measuring and monitoring the burden of cancer, cancer registries will become an invaluable source of evidence and guidance for policy setting, program implementation, and improving practice. Finally, it is believed that a cost-effective strategy for managing CRC will be to implement policies that educate people about the health consequences of poor diet and lifestyle behaviors that are linked to CRC. Screening for CRC is thought to be cost-effective in high-income countries, however it might not be for low and middle income countries where the incidence rate is lower and where the implementation of a screening program will require the purchase and maintenance of expensive equipment, skilled specialists, and education of the public (31).
