# Supplementary material for: Estimating the incidence of colorectal cancer in South East Asia
Source: Croat Med J. 2013 Dec;54(6):532–40. doi: 10.3325/cmj.2013.54.532 (PMC3893985; doi:10.3325/cmj.2013.54.532)
Supplement: Supplementary Table 1 [file CroatMedJ_54_s001.pdf]

Supplementary table 1 Search items for the systematic data extraction from published papers

1	Prevalence/ or prevalen*.tw
2	Mortality/ or mortal*.tw
3	Global burden of disease/ or (disease adj3 burden*).tw
4	Incidence/ or inciden*.tw
5	1 or 2 or 3 or 4
6	colorectal adenoma/ or CRC/ or colon cancer/ or rectum cancer/ or colorectal carcinoma/ or colorectal disease
7	(bowel* or large intestine* or large bowel* or gut* or colorect* or colo* or rect*) adj3 (maligna* or carcinoma* or neoplas* or cancer* or tumo* or polyp*).tw
8	6 or 7
9	Asia, southeastern/ or (Asia* adj3 South-East*).tw or (Asia* adj3 South* adj3 East*).tw
10	Exp Bangladesh/ or exp Bhutan/ or exp Korea/ or exp India/ or exp Indonesia/ or exp Maldives/ or exp Myanmar/ or exp Nepal/ or exp Sri Lanka/ or exp Thailan/ or exp Timor-Leste/
11	(India* or Myanmar or Korea or Bangladesh or Bhutan or Indonesia or Maldives or Nepal or Sri Lanka or Thailand or Timor).tw
12	9 or 10 or 11
13	5 and 8 and 12
14	Limit 13 to yr = 1980-current
