# Supplementary figures and images for: Estimating the incidence of colorectal cancer in South East Asia
Source: Croat Med J. 2013 Dec;54(6):532–40. doi: 10.3325/cmj.2013.54.532 (PMC3893985; doi:10.3325/cmj.2013.54.532)

**Supplementary figure 1** Sources of data

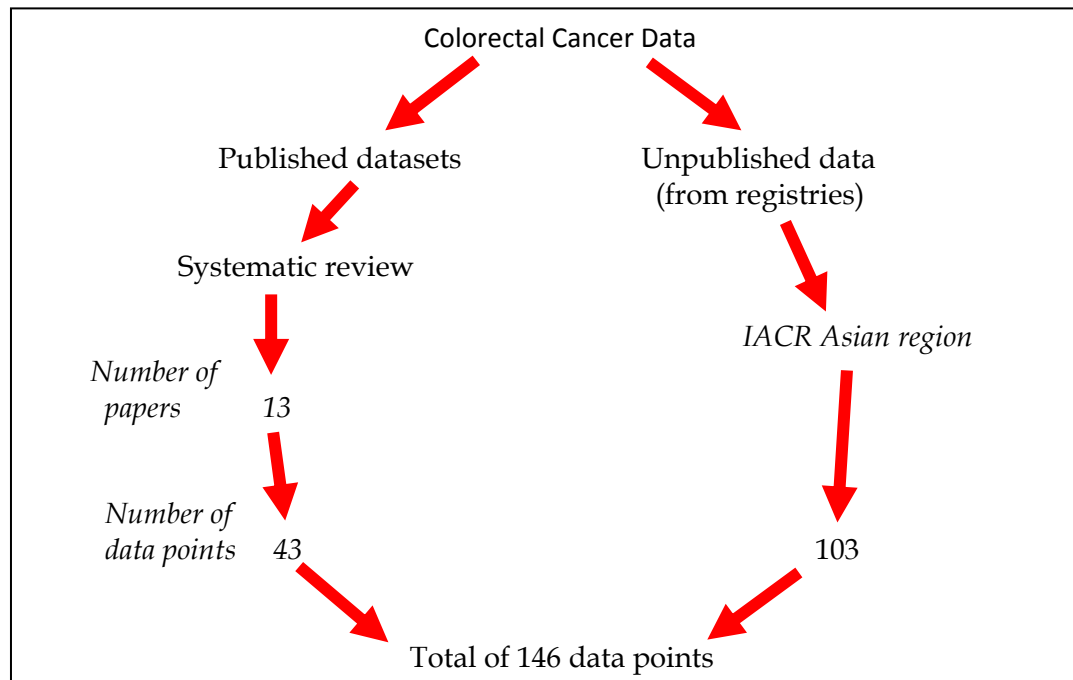

Supplement: Supplementary Figure 1 [file CroatMedJ_54_s002.pdf]

**Supplementary figure 3** Histogram of the years of study of the included data points.

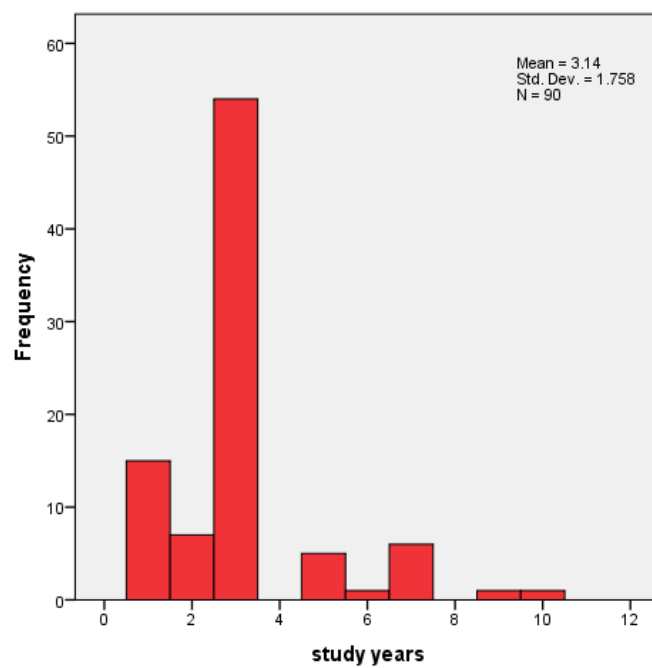

Supplement: Supplementary Figure 3 [file CroatMedJ_54_s004.pdf]

Supplementary figure 6: Meta-analysis of the incidence rate of colon cancer

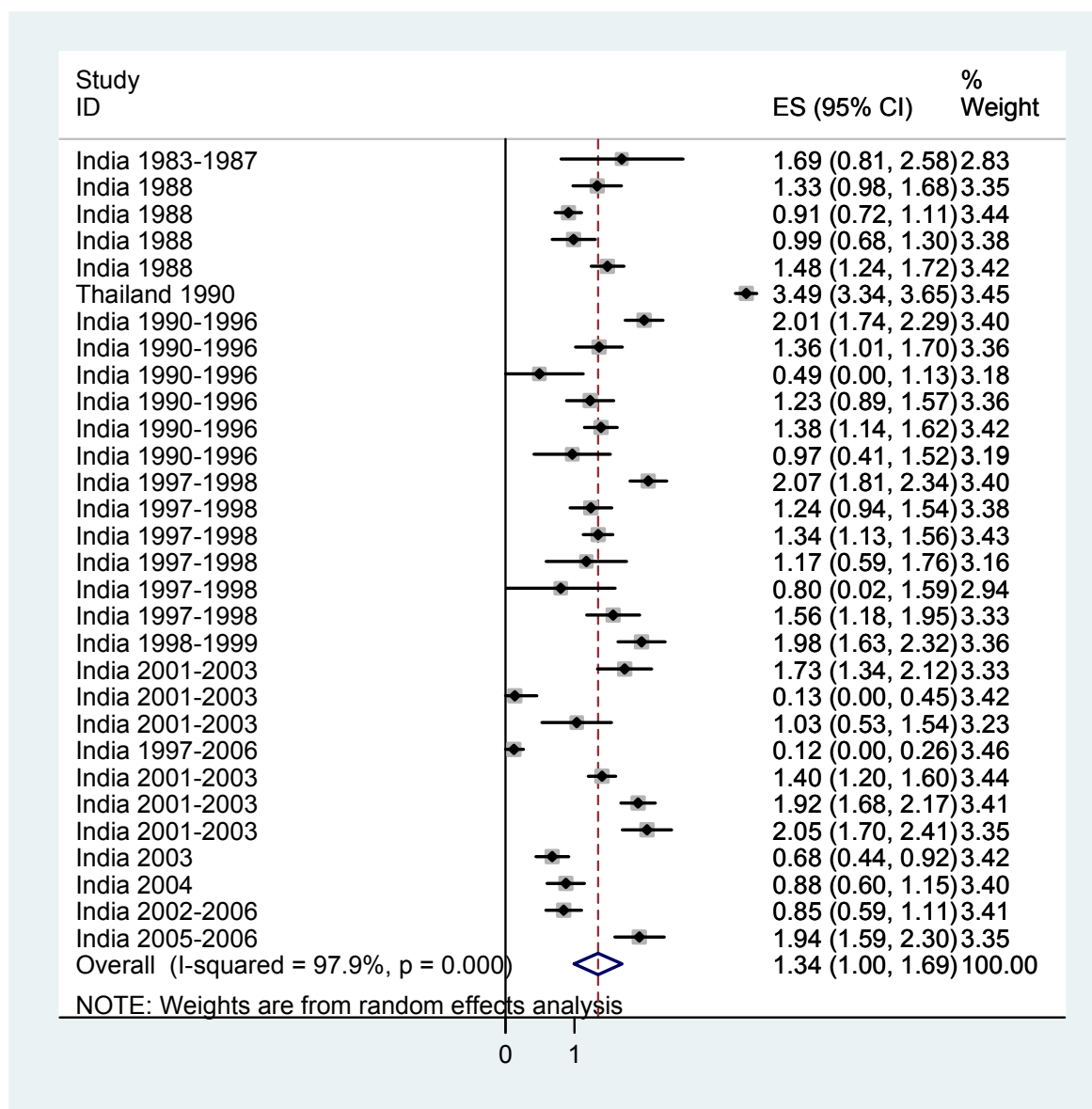

Supplement: Supplementary Figure 6 [file CroatMedJ_54_s008.pdf]

Supplementary figure 7: Meta-analysis of the incidence rate of rectal cancer.

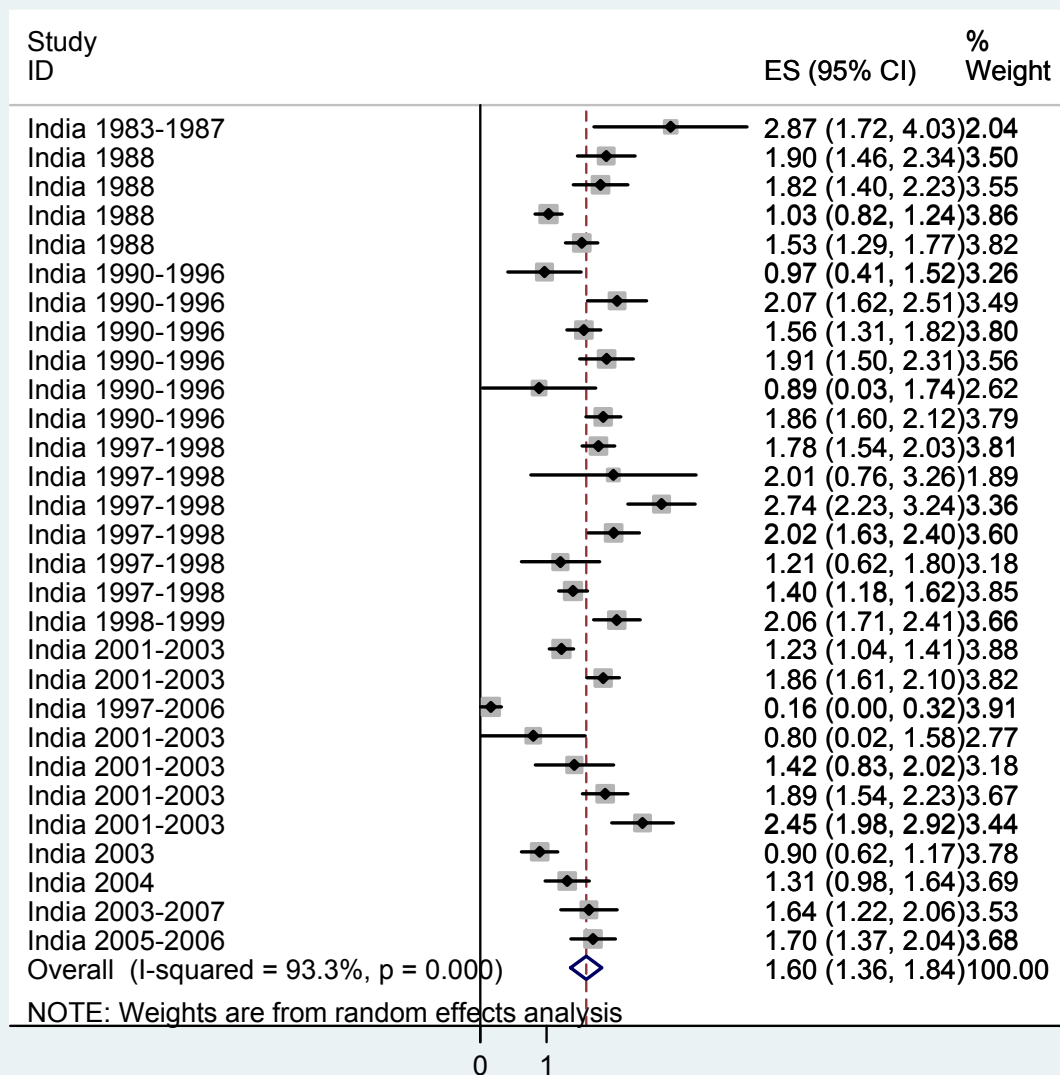

Supplement: Supplementary Figure 7 [file CroatMedJ_54_s009.pdf]
