# Supplementary material for: Estimating the incidence of colorectal cancer in South East Asia
Source: Croat Med J. 2013 Dec;54(6):532–40. doi: 10.3325/cmj.2013.54.532 (PMC3893985; doi:10.3325/cmj.2013.54.532)

Supplementary figure 2 Number of data sets by the mean year of each study, regarding the publication type or the data source.

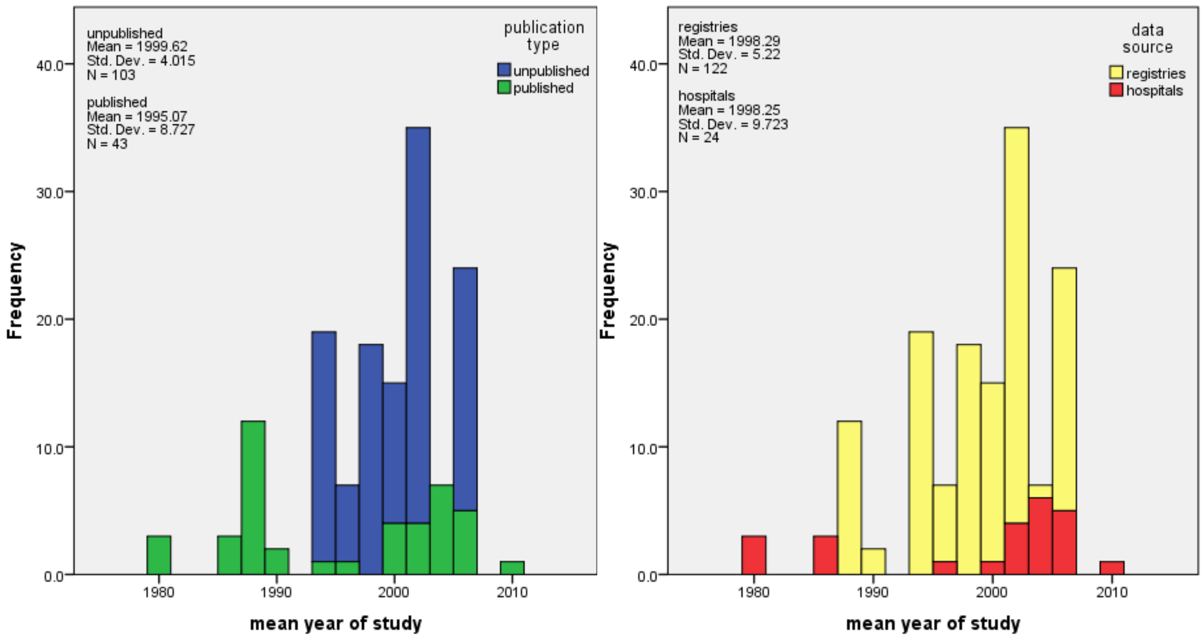

Supplement: Supplementary Figure 2 [file CroatMedJ_54_s003.pdf]
