# Supplementary material for: Estimating the incidence of colorectal cancer in South East Asia
Source: Croat Med J. 2013 Dec;54(6):532–40. doi: 10.3325/cmj.2013.54.532 (PMC3893985; doi:10.3325/cmj.2013.54.532)

Supplementary figure 5 Histogram of the colorectal cancer incidence rate per year per 100,000 population.

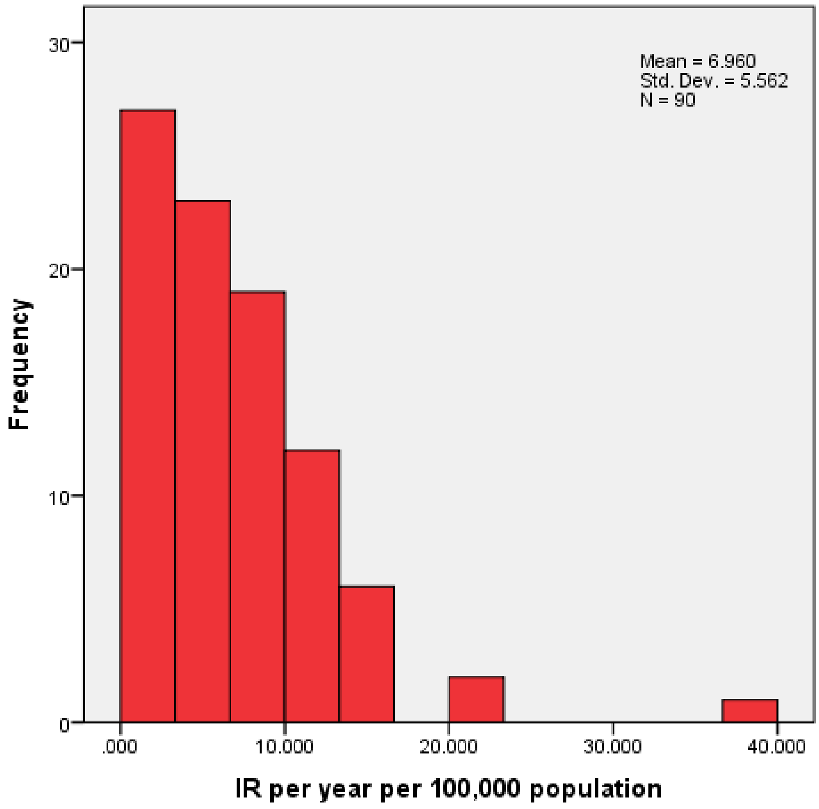

Supplement: Supplementary Figure 5 [file CroatMedJ_54_s006.pdf]
