# Supplementary material for: Estimating the incidence of colorectal cancer in South East Asia
Source: Croat Med J. 2013 Dec;54(6):532–40. doi: 10.3325/cmj.2013.54.532 (PMC3893985; doi:10.3325/cmj.2013.54.532)
Supplement: Supplementary Table 2 [file CroatMedJ_54_s007.pdf]

Supplementary table 2 Statistics for the crude annual incident rate of colon and rectal cancer (per year per 100,000 population), the number of cases and the population size. The number of data points is denoted by N. (SD: standard deviation, IR: incidence rate, CI: confidence interval)

		Number of cases		Population		IR		IR	
**Colon cancer**	**N**	**mean (SD)**	**median ($Q_1 – Q_3$)**	**mean (SD)**	**median ($Q_1 – Q_3$)**	**mean (SD)**	**median (Q_1 - Q_3)**	**Meta-analysis (95% CI)**	**I^2 (95% CI)**
**All data**	30	306.3 (438.97)	128 (39.5 – 392.25)	7,098,564 (9730662)	4,572,876 (2,279,323 – 9,546,955)	1.34 (0.67)	1.34 (0.90 – 1.78)	1.35 (1.00 – 1.69)	98% (98 – 98)
Publication status									
Systematic review	12	247.67 (526.53)	78 (38.5 – 189)	9,279,699 (14,469,435)	4,686,438 (3,913,547 – 8,672,761)	1.36 (0.86)	1.16 (0.85 – 1.88)	1.36 (0.65 – 2.07)	99% (99 – 99)
Cancer registries	18	345.39 (381.16)	262.5 (43.75 – 493.5)	5,644,473 (4,522,690)	4,376,217 (1,292,974 – 10,613,950)	1.33 (0.53)	1.35 (1.02 – 1.78)	1.35 (1.08 – 1.62)	92 (89 – 94)
*p-value**						0.898	0.611		
Data source									
Hospital based	5	57 (35.16)	40 (31 – 91.50)	3,661,871 (1,975,416)	4,572,876 (1,675,783 – 5,192,456)	1.06 (0.75)	0.88 (0.40 – 1.82)	1.01 (0.38 – 1.65)	96% (94 – 98)
Cancer registries	25	356.16 (465.93)	203 (51.50 – 444)	7,785,902 (10,526,580)	4,800,000 (2,694,236 – 10,170,183)	1.40 (0.66)	1.34 (0.98 – 1.83)	1.41 (1.06 – 1.76)	97% (97 – 98)
*p-value**						0.315	0.303		
**Rectal cancer**	**N**	**mean (SD)**	**median ($Q_1 – Q_3$)**	**mean (SD)**	**median ($Q_1 – Q_3$)**	**mean (SD)**	**median (Q_1 - Q_3)**	**Meta-analysis (95% CI)**	**I^2 (95% CI)**
**All data**	29	278.48 (317.15)	152 (63 – 375.5)	5,419,204 (3,895,375)	4,452,434 (2,035,241 – 9,353,548)	1.62 (0.59)	1.70 (1.22 – 1.96)	1.60 (1.36 – 1.84)	93% (91 – 95)
Publication status									
Systematic	11	119	97	5,050,581	4,572,876	1.54	1.64	1.48	96%

review		(84.39)	(60 – 152)	(2,733,715)	(3,500,000 – 6,429,112)	(0.70)	(1.03 – 1.9)	(1.04 – 1.92)	(94 – 97)
Cancer registries	18	375.94 (367.37)	315.5 (57.5 – 582)	5,644,473 (4,522,690)	4,376,217 (1,292,974 – 10,613,950)	1.67 (0.53)	1.82 (1.22 – 2.01)	1.70 (1.50 – 1.89)	81% (70 – 87)
*p-value**						0.573	0.590		
Data source									
Hospital based	6	107.83 (93.3)	79.5 (41 – 161)	3,634,892 (1,768,102)	4,036,438 (2,099,594 – 4,882,666)	1.43 (0.91)	1.48 (0.71 – 2.00)	1.34 (0.68 – 1.99)	96% (94 – 98)
Cancer registries	23	323 (340.62)	224 (73 – 507)	5,884,676 (4,184,135)	4,452,434 (1,547,076 – 9,925,891)	1.67 (0.50)	1.82 (1.23 – 2.01)	1.68 (1.51 – 1.86)	83% (75 – 88)
*p-value**						0.390	0.333		

* P-values were calculated using t-test for mean values and Mann-Whitney test for median values
