# Supplementary material for: Estimating the incidence of colorectal cancer in South East Asia
Source: Croat Med J. 2013 Dec;54(6):532–40. doi: 10.3325/cmj.2013.54.532 (PMC3893985; doi:10.3325/cmj.2013.54.532)

Supplementary figure 9 Boxplots of the incident rate (per year per 100000 population) of colorectal cancer by age groups.

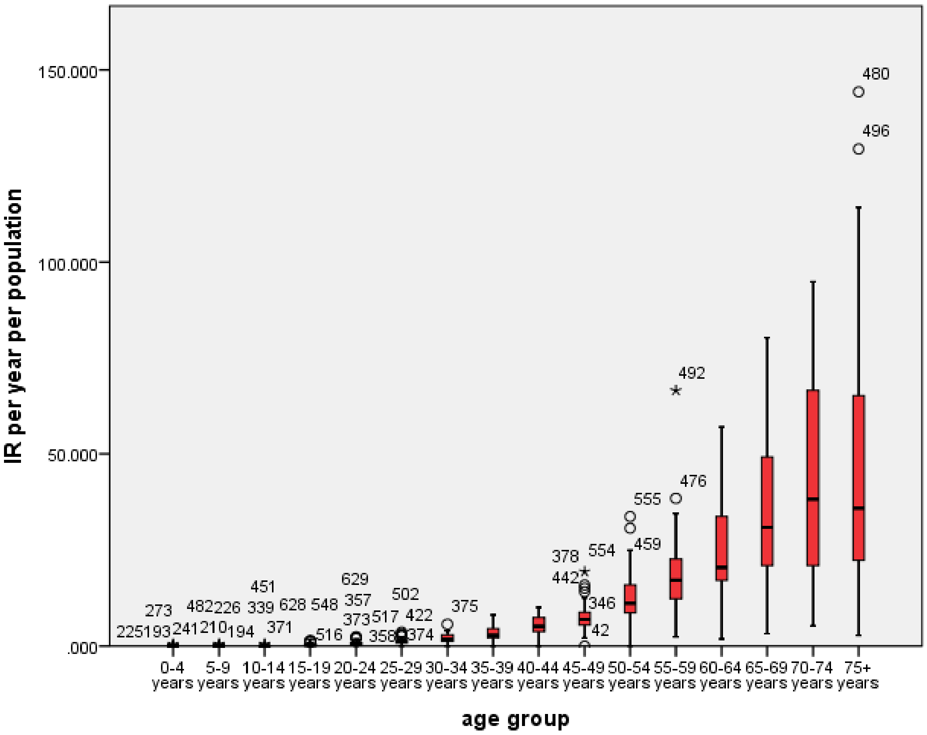

Supplement: Supplementary Figure 9 [file CroatMedJ_54_s011.pdf]
